# Association of 25(OH)-Vitamin D3 Serum Concentrations and Vitamin D Receptor Gene Variants with the Risk of Idiopathic Spontaneous Preterm Birth in the Croatian Population

**DOI:** 10.3390/ijms252111712

**Published:** 2024-10-31

**Authors:** Milena Gašparović Krpina, Sanja Dević Pavlić, Tea Mladenić, Merica Aralica, Anita Barišić, Alemka Brnčić-Fischer, Saša Ostojić, Nina Pereza

**Affiliations:** 1 Croatia Policlinics, Vukovarska Ulica 7A, 51000 Rijeka, Croatia; milenakrpina@gmail.com; 2 Department of Medical Biology and Genetics, Faculty of Medicine, University of Rijeka, Braće Branchetta 20, 51000 Rijeka, Croatia; sanja.devic@uniri.hr (S.D.P.); tea.mladenic@uniri.hr (T.M.); sasa.ostojic@uniri.hr (S.O.); 3 Clinical Department of Laboratory Diagnostics, Clinical Hospital Center Rijeka, Vjekoslava Dukića 7, 51000 Rijeka, Croatia; merica.aralica@gmail.com; 4 Clinics for Gynecology and Obstetrics, Clinical Hospital Center Rijeka, Vjekoslava Dukića 7, 51000 Rijeka, Croatia; anita.barisic@uniri.hr (A.B.); alemkabf@uniri.hr (A.B.-F.)

**Keywords:** 25(OH)-vitamin D3, polymorphisms, preterm birth, vitamin D receptor gene

## Abstract

Preterm birth (PTB) forms a heterogeneous group with possible genetic predisposition. 25(OH)-vitamin D3 plays a significant role during implantation, placentation, and the maintenance of normal pregnancy. The aim of our research was to examine whether FokI, Cdx2, and ApaI *VDR* gene variants, as well as serum concentrations of 25-hydroxy25(OH)-vitamin D3 in women and their newborns, might be predisposing factors for idiopathic spontaneous preterm birth. The patient group consisted of 44 pairs of women with ISPTB and their children, and the control group consisted of 44 pairs of women who delivered at term and their children. At the time of delivery, peripheral blood was collected from every woman, and after newborn delivery, umbilical cord blood was collected. For genotyping of the rs2228570 C/T, rs11568820 G/A, and rs7975232 T/G SNPs, a combination of polymerase chain reaction (PCR) and restriction fragment length polymorphism (RFLP) was used. Serum concentrations of 25(OH)-vitamin D3 were determined using high-performance liquid chromatography (HPLC). There were no statistically significant differences in the frequencies of *VDR* genotypes and alleles between women with ISPTB and control women. There was a statistically significant difference in the distribution of *VDR* Cdx-2 (rs11568820) genotypes between preterm-born children and controls, with the GG genotype and G allele being more prevalent among patients than controls (*p* < 0.001). There were no statistically significant differences in mean values between women with ISPTB and control women, nor between preterm and full-term newborns, although the 25(OH)-vitamin D3 concentrations in preterm-born children were lower than in controls. Furthermore, there was a statistically significant correlation in 25(OH)-vitamin D3 concentrations between mothers and children both in the patient and in the control groups (b = 0.771, *p* < 0.001). The results of our study demonstrate a notable association between the *VDR* Cdx2 gene polymorphism and idiopathic spontaneous preterm birth (ISPTB) in a Caucasian population, but because of the small number of participants, further research is needed.

## 1. Introduction

Preterm birth (PTB) is defined as the birth occurring before the 37th completed week of gestation, with an overall incidence of 11% worldwide [1]. Depending on the week of gestation, PTB is divided into three categories: late (32–36 weeks), moderate (28–31 weeks), and very early (˂28 weeks) [2,3]. Furthermore, preterm births are classified as spontaneous (SPTB) and medically induced. The SPTBs form a heterogeneous group, in which the causes may include multiple pregnancies, urogenital infections, uteroplacental ischemia, or bleeding. By excluding known causes of PTB, the remaining SPTBs fall into the category of PTB of unknown etiology, or idiopathic SPTB (ISPTB), which account for almost half of all cases.

Given the frequent hospitalizations of preterm infants in intensive care units and their potential long-term health consequences, as well as a high mortality rate, various factors have been studied for their potential contribution to the causes and susceptibility to ISPTB, as well as identification of potential biomarkers. Numerous epidemiological studies suggest a possible genetic predisposition for ISPTB [4,5]. For instance, the strongest predictor for ISPTB is a personal or family history of PTB [6]. In addition, studies on twins indicate a heritability of 20 to 40% [7], whereas the incidence of PTB varies significantly depending on ethnic and racial background. Among the susceptibility factors that have been studied in ISPTB but never reached unambiguous conclusions on causative association is vitamin D deficiency and the potential role of vitamin D receptor (VDR) gene variants.

25(OH)-vitamin D3 plays a significant role during implantation, placentation, and the maintenance of normal pregnancy. 25(OH)-vitamin D3 insufficiency affects almost 50% of the global population and more than 66% of pregnant women [8]. Additionally, inadequate serum concentrations of 25(OH)-vitamin D3 in pregnant women are associated with numerous pregnancy complications and poorer perinatal outcomes, such as gestational diabetes mellitus, preeclampsia, primary cesarean section, and intrauterine growth restriction (IUIGR) [9,10,11,12,13]. Inadequate 25(OH)-vitamin D3 status is also associated with a higher risk of chronic diseases, as well as cancer, autoimmune and inflammatory diseases, type 2 diabetes mellitus, neurocognitive disorders, and mortality [14].

25(OH)-vitamin D3 is a fat-soluble vitamin and prohormone. Its two main forms are D2 (ergocalciferol), the source of which is plant-based nutrition, and D3 (cholecalciferol), which is mainly synthesized in skin cells after exposure to UVB radiation. In addition to promoting calcium absorption from the intestine and regulating calcium and phosphate homeostasis, novel actions of 25(OH)-vitamin D3 include its effects on cell proliferation, differentiation, and conducting crucial roles in immune system responses [15]. 25(OH)-vitamin D3 deficiency is considered for concentrations below 20 ng/mL (or 50 nmol/L), insufficiency between 20 and 30 ng/mL (50 and 75 nmol/L), and concentrations above 30 ng/mL are considered satisfactory [16].

The results of previous individual studies on the association between 25(OH)-vitamin D3 levels and the risk of PTB are conflicting, which could at least partially be explained by inconsistencies and differences in the inclusion and exclusion criteria for participants, as well as laboratory methods for detecting 25(OH)-vitamin D3. However, meta-analyses suggest that pregnant women with inadequate serum levels of 25(OH)-vitamin D3 might have an increased risk of PTB [17,18,19], whereas the results of randomized controlled trials indicate that 25(OH)-vitamin D3 supplementation reduces the risk of PTB [20].

The metabolic effect of 25(OH)-vitamin D3 is achieved through its binding to VDR, which is encoded by the *VDR* gene. The *VDR* gene is located on chromosome 12, and more than 60 sequence variants have been discovered, among which the most studied include the single nucleotide polymorphisms (SNP): rs11568820 (Cdx2), rs2228570 (FokI), rs1544410 (BsmI), rs7975232 (ApaI), and rs731236 (TaqI) [21]. These SNPs are associated with alterations in gene expression levels, structure, and/or function of the VDR mRNA and protein. Given that VDR is significantly expressed at the maternal-fetal interface, *VDR* gene variants may have a specific impact on the course of pregnancy. The association of these SNPs in the *VDR* gene with SPTB in mothers has been examined in only a few genetic association studies, and the results have been conflicting [22,23,24,25,26,27]. To the best of our knowledge, the association between *VDR* gene SNPs and serum 25(OH)-vitamin D3 levels in PTB has been studied only in one study [28].

Therefore, the aim of our research was to examine whether FokI, Cdx2, and ApaI *VDR* gene variants, as well as serum concentrations of 25-hydroxy25(OH)-vitamin D3 in women and their newborns, individually or in combination, might be predisposing factors for ISPTB. In the present study, we focused on strict inclusion and exclusion criteria, as well as the European population in the Republic of Croatia.

## 2. Results

### 2.1. Epidemiological Data

Epidemiological data of women and newborns with analyzed parameters are presented in Table 1. Statistically significant differences between the ISPTB group and the control group were found for newborn gender, gestational age, birth weight, and length (*p* < 0.001).

### 2.2. Genetic Association Study

Table 2 shows the distribution of *VDR* FokI (rs2228570), ApaI (rs7975232), and Cdx2 (rs11568820) genotypes and alleles in both patient and control groups. There were no statistically significant differences in the frequencies of *VDR* genotypes and alleles between women with ISPTB and control women. In addition, there were no statistically significant differences in the distribution of *VDR* FokI and ApaI genotypes and alleles between preterm-born children and controls. However, there was a statistically significant difference in the distribution of *VDR* Cdx-2 (rs11568820) genotypes between preterm-born children and controls, with the GG genotype and G allele being more prevalent among patients than controls (*p* < 0.001). Associations between *VDR* FokI and ApaI genotypes and alleles with ISPTB did not reach statistical significance (ORs and 95% CI), whereas the presence of the G allele in newborns increased the risk for ISPTB 3.63 times (95% CI = 1.88–7.01, *p* < 0.001). Due to the limited number of samples in the study group, multiple genotype and haplotype combinations analysis was not performed.

### 2.3. Biochemical Study

Vitamin D deficiency was present in 7 women with ISPTB (15.9%), 13 control women (29.5%), and 23 newborns with ISPTB and controls (52.3%), each. In addition, vitamin D insufficiency was present in 11 women with ISPTB (25%), 9 control women (20.5%), 16 newborns with ISPTB (36.4%), and 8 controls (18.2%). There were no statistically significant differences between women or newborn groups of participants (*p* = 0.311 and *p* = 0.05, respectively).

Table 3 shows the serum concentrations of the 25(OH)-vitamin D3 in both patients and control groups. There were no statistically significant differences in mean values between women with ISPTB and control women, nor between preterm and full-term newborns, although the 25(OH)-vitamin D3 concentrations in preterm-born children were lower than in controls. Furthermore, there was a statistically significant correlation in 25(OH)-vitamin D3 concentrations between mothers and children both in the patient and in the control groups (b = 0.771, *p* < 0.001). Women with higher 25(OH)-vitamin D3 concentrations gave birth to children with higher concentrations, and women with lower concentrations gave birth to children with lower concentrations.

### 2.4. Associations Between VDR SNPs and 25(OH)-Vitamin D3 Serum Concentrations

There were no statistically significant differences between individual *VDR* FokI and ApaI genotypes and 25(OH)-vitamin D3 concentrations in the patient and control groups. On the other hand, the AA genotype of *VDR* Cdx-2 SNP had significantly higher 25(OH)-vitamin D3 concentrations in mothers both in the patient and control groups (*p* = 0.01, *p* = 0.001, respectively), but not in children. However, when all three gene variants were tested for their correlation with 25(OH)-vitamin D3 concentrations in combination, there were no correlations for any genotype combination in women and children in the patient and control groups. In addition, there were no correlations between all three gene variants, 25(OH)-vitamin D3 concentrations, and clinical characteristics, including maternal BMI, gestational age at delivery, newborn birth weight, or newborn birth length (all *p* > 0.05).

## 3. Discussion

In our study, we analyzed the association of 25(OH)-vitamin D3 serum concentrations and vitamin D receptor gene variants with the risk of idiopathic spontaneous preterm birth in the Croatian population. The obtained results revealed that the GG genotype of the *VDR* Cdx2 polymorphism was significantly more frequent in preterm-born children than controls, although the levels of 25(OH)-vitamin D3.

The role of 25(OH)-vitamin D3 in pregnancy outcomes has been intensively studied over the past two decades, especially due to its important immunomodulatory and antimicrobial role at the maternal-fetal interface [29,30,31]. The concentration of the active form of 25(OH)-vitamin D3 in circulation continuously increases during pregnancy, reaching double to triple the concentration at term compared to non-pregnant women. The concentration of 25(OH)-vitamin D3 in the fetus is entirely dependent on the status of maternal circulating 25(OH)-vitamin D3, and inadequate concentrations in pregnant women may influence pregnancy outcomes and newborn health [32]. In addition, circulating levels of 25(OH)-vitamin D3 might also be influenced by the presence of certain *VDR* gene variants. The VDR belongs to the superfamily of nuclear receptors and, as a transcription factor, is an important mediator in the metabolic pathway of 25(OH)-vitamin D3. VDR promotes transcription of downstream genes, the products of which enhance calcium absorption, hormone secretion, cell migration, differentiation, and proliferation [33,34].

In our research, the GG genotype of the *VDR* Cdx2 polymorphism was significantly more frequent in preterm-born children than in controls. The Cdx2 *VDR* gene polymorphism has been investigated in numerous diseases, but the results of different studies have been inconsistent. For example, Serrano et al. demonstrated an association between Cdx2 polymorphism and risk of cancer in a meta-analysis of 25 studies (17,425 patients) [35]. Vladoiu et al. have shown that the AA genotype is more common among patients with male infertility than among controls [36]. On the other hand, our research is in line with AbdElneam et al., who investigated the association between Cdx2 and FokI *VDR* polymorphism and serum levels of 25(OH)-vitamin D3 in patients with psoriasis. They found that the Cdx2 GG genotype is more prevalent among patients than among controls [37]. Similar findings were reported by Torkko et al. in 2008, where GG genotypes were shown to increase the risk of prostate cancer in Hispanic White men [38]. Iqbal et al. demonstrated an association between the GG genotype and breast cancer, while Rehman showed that AG and GG genotypes had a significant association with various types of cancers [39,40].

The G allele was previously associated with 30% less transcriptional activity compared to the A allele [41], and our study further confirmed that the Cdx-2 AA genotype is associated with higher concentrations of 25(OH)-vitamin D3 in the control groups of both mothers and children but not the patient groups. Similar to our study, Gwenzi et al. examined 25(OH)-vitamin D3 status and the Cdx2 genotype in colorectal cancer [42]. As many as 65% of patients had the GG genotype for Cdx2, and in their study, serum 25(OH)-vitamin D3 levels did not differ according to the Cdx2 genotype. Additionally, like in our research, Gwenzi and colleagues did not confirm that the AA and AG genotypes for Cdx2 were associated with better survival in colorectal cancer. Therefore, further research on the association between the *VDR* Cdx-2 gene variant, 25(OH)-vitamin D3 concentrations and ISPTB is necessary, especially in larger samples.

The association of other *VDR* SNPs in mothers and children has been investigated in only a few genetic association studies, and the results are conflicting. Manzon et al. demonstrated in a study of 33 mothers with preterm birth and their preterm infants that the C allele of the FokI SNP is more frequent, while the T allele of the TaqI is less frequent among mothers with PTB compared to controls [24]. Baczynska-Strzecha et al. showed that certain combinations of BsmI-ApaI-TaqI genotypes are significantly higher in women with PTB, while the distribution of individual genotypes did not differ between patients and controls [28]. In contrast, Rosenfield et al. demonstrated that mothers who are homozygous for the VDR Apa (AA) genotype (which corresponds to our GG) have an increased risk of PTB [29]. Javorski et al. investigated the association of FokI and Cdx2 SNPs with PTB, but contrary to our research, the AA genotype for Cdx2 is statistically more significant in PTB cases [27]. Barchitta et al. showed in a study of 187 pairs of mothers and their preterm infants that the AA genotype of the FokI SNP in mothers is associated with an elevated risk of preterm birth [25].

The influence of *VDR* gene polymorphisms on serum 25(OH)-vitamin D3 levels is still under investigation, but numerous studies in other disorders suggest that the genetic structure of the *VDR* gene affects serum concentrations of 25(OH)-vitamin D3 [43]. The association between *VDR* gene polymorphisms and serum 25(OH)-vitamin D3 levels has been studied in various pathological conditions, such as vitiligo, lumbar disc degeneration, and autism [44,45,46].

To the best of our knowledge, the association of SNPs in the *VDR* gene and serum 25(OH)-vitamin D3 levels in PTB has been studied only in a Brazilian population in the research conducted by Dutra et al. [30]. The difference between our study and that of Dutra et al. is that our participants were carefully selected patients with idiopathic spontaneous preterm births, while in Dutra’s study, the exclusion factors included neonates with significant malformations, genetic syndromes, or severe neonatal anoxia, as well as cases where measuring 25(OH)-vitamin D3 was not possible. In their research group, as much as 45% of participants had hypertensive diseases in pregnancy, 5% had gestational diabetes, and 22.5% had urinary tract infections. In our group, all of these conditions were exclusion factors.

In the study by Dutra et al., it was demonstrated that certain Taq and BsmI genotypes in mothers and specific haplotype combinations were more common among mothers with PTB and that certain Taq, Apa, and Fok genotypes resulted in lower 25(OH)-vitamin D3 levels. Certain genotypes of BsmI and ApaI are associated with 25(OH)-vitamin D3 deficiency and have a higher risk for preterm birth (OR 2.36 and 7.99). Among preterm infants, certain BsmI, Apa, and Fok genotypes and haplotype combinations were also more frequently present, with children having certain Fok genotypes showing lower 25(OH)-vitamin D3 levels.

In our study, the concentration of 25(OH)-vitamin D3 in preterm infants is lower than in controls, although the difference is not statistically significant; however, there is a statistically significant correlation in 25(OH)-vitamin D3 concentrations between mothers and their children.

The advantage of our study is the strict exclusion criteria, as our participants comprise ISPTB, meaning all known causes of preterm birth have been excluded. Additionally, in our study we utilized the HPLC method, the golden standard for 25(OH)-vitamin D3 analysis, while in Dutra’s study, serum 25(OH)-vitamin D3 levels were determined using electrochemiluminescence.

The possible limitations of our study may lie in the small number of participants. Increasing the sample size would provide greater statistical power and more reliable results. The strengths of our study include the careful selection of the control group to match the characteristics of the patient group as closely as possible, significantly reducing the likelihood of variations in population parameters influencing the study outcomes. Likewise, replicating the study in different ethnic groups or geographic regions may help to confirm the findings and determine whether they are specific to this population or more broadly applicable. This study can have clinical implications regarding the role of VDR polymorphism and vitamin D concentration in the prediction of PTB, but further and larger studies are needed to confirm these findings.

## 4. Materials and Methods

### 4.1. Patients

This retrospective case-control study enrolled pairs of women who gave birth at the Clinics for Gynecology and Obstetrics, Clinical Hospital Center Rijeka, Croatia, and their newborns between 2017 and 2019. The number of births per year in the Clinical Hospital Centre Rijeka was: 2520 (in 2017), 2474 (in 2018), and 2381 (in 2019). All participants were informed about the study, purpose, and methodology, and their consent to participate was confirmed by providing written informed consent. The patients are part of the TransMedRi Biobank—a biorepository of samples within the preterm birth research. The study was approved by the Ethical Committee for Biomedical Research of the Faculty of Medicine in Rijeka, Croatia (003-08/19-01/5; 2170-24-24-3-19-3) and the Ethics Committee of the Clinical Hospital Center Rijeka (003-05/19-1/21; 2170-29-02/1-19-2).

The study included two groups of participants: patients and controls. Structured questionnaires were used to collect epidemiological and clinical data in both groups, which were completed through interviews with the participants on the day of delivery.

The patient group consisted of 44 pairs of women with ISPTB and their children. The inclusion criteria were defined as delivery before the 37th week of gestation that began spontaneously and was not medically induced. Exclusion criteria included conception through assisted reproduction, multiple pregnancies, previous cervical procedures (e.g., conization and large loop excision of the transformation zone), lower reproductive tract infection, pregnancy complications (hypertension in pregnancy, gestational diabetes mellitus), stillbirth, congenital anomalies, or confirmed infections in the newborn.

The control group consisted of 44 pairs of women who delivered at term and their children. The inclusion criteria for term delivery were defined as delivery between the 37th and 42nd weeks of gestation. The exclusion criteria were all pregnancy-related diseases such as (pre)eclampsia, gestational diabetes, urogenital infections, fetal malformations, conception through assisted reproduction, previous cervical procedures, multiple pregnancies, stillbirth, as well as a previous history of preterm birth (PTB) and confirmed infections in the newborn. All deliveries were completed naturally after an uncomplicated pregnancy. The small sample size is a reflection of the fact that two scientific projects that funded the sample collection lasted in the period between 2017 and 2019.

Patients and controls were matched based on parity, age, ethnicity, socio-economic and demographic status, place of residence, antenatal care, access to tertiary medical care, and season of delivery, ensuring that controls gave birth during the same season as patients with spontaneous PTB.

In both the patient and control groups, gestational age was determined by the first day of the last menstrual cycle and confirmed by ultrasound in the first trimester. If the due date differed between the first day of the last menstrual cycle and the ultrasound findings, gestational age was adjusted according to the ultrasound results.

### 4.2. Sample Collection

At the time of delivery, 10 mL of peripheral blood was collected from every woman by peripheral venepuncture into tubes containing ethylenediaminetetraacetic acid (EDTA) and tubes containing a clot-separating gel.

After newborn delivery, but before the delivery of the placenta, 10 mL of umbilical cord blood was collected into a tube containing EDTA and a tube containing a clot-separating gel. After collection, the tubes for DNA extraction were stored in a refrigerator at +4 °C for further procedure. The tubes for biochemical analysis of serum 25(OH)-vitamin D3 concentrations were centrifuged (1500 rpm for 10 min); the serum was aliquoted into microtubes and frozen at −20 °C.

### 4.3. Genetic Association Study

In the genetic association study, genomic DNA was isolated from maternal peripheral and newborn cord blood samples. Genomic DNA isolation was carried out at the Department of Medical Biology and Genetics at the Faculty of Medicine, University of Rijeka, Croatia, using commercially available kits and following the manufacturer’s protocol (Qiagen FlexiGene DNA kit, Qiagen GmbH, Hilden, Germany). The concentration and purity of the isolated genomic DNA were determined using a spectrophotometer (Nanodrop 2000, Thermo Fisher Scientific, Wilmington, DE, USA). Blood samples and genomic DNA were stored at −20 °C at the department until further analysis.

For genotyping of the rs2228570 C/T, rs11568820 G/A, and rs7975232 T/G SNPs, a combination of polymerase chain reaction (PCR) and restriction fragment length polymorphism (RFLP) was used. The conditions for the PCR-RFLP reactions, including primers, thermal cycles, and types of restriction endonucleases, were taken from our previously published study [47].

### 4.4. Biochemical Analysis of Serum 25(OH)-Vitamin D3 Concentration

The biochemical analysis of serum 25(OH)-vitamin D3 concentration was conducted at the Clinical Department of Laboratory Diagnostics, Clinical Hospital Center Rijeka. Serum concentrations of 25(OH)-vitamin D3 were determined using the high-performance liquid chromatography (HPLC) on the Nexera X2 (Shimadzu, Kyoto, Japan).

Sample preparation was done manually, using components from the 25(OH)-vitamin D3 commercial HPLC kit (Recipe, Munchen, Germany). Briefly, 500 μL Precipitat *p* was mixed with 400 μL serum following 400 μL internal standard for 30 s on the vortex mixer. After mixing, sample was centrifuged for 5 min at 10,000× *g*, generating two liquid phases. The upper liquid phase of supernatant was used for HPLC analysis.

Prepared samples were analyzed at the Nexera X2 modular ultra-high liquid performance chromatograph using 25(OH)-vitamin D3, commercial HPLC kit (Recipe, Munchen, Germany). The Nexera X2 was equipped with two LC-30AD Solvent Delivery Units, SIL-30A Autosampler, CTO-30A Column Oven, and SPD-M30A Photodiode Array Detector. The HPLC analysis conditions were as follows: low rate 0.9 mL/min, temperature 40 °C, injection volume 30 μL, run time 12 min, and the detector set at 264 nm. Linearity and limit of quantitation were 7.3–1210 nmol/L and 2.7 nmol/L for 25(OH)-vitamin D3 as well as 6.5–1250 nmol/L.

### 4.5. Statistical Analysis

Statistical analysis was performed in Statistica for Windows, version 14.0.0.15 (StatSoft, Inc., Tulsa, OK, USA). Statistical significance was calculated using the GAS Power Calculator: https://csg.sph.umich.edu/abecasis/cats/gas_power_calculator/ (accessed on 20 September 2024). Hardy-Weinberg analysis was performed to compare the observed and expected genotype frequencies using the Simple Hardy-Weinberg Calculator-Court Lab (Washington State University College of Veterinary Medicine, Pullman, WA, USA). Descriptive statistics were used for epidemiological data.

Differences in allele and genotype frequencies of VDR gene variants and alcohol and tobacco use between patients and controls were determined using the Pearson Chi-square test. The odds ratio (OR) and associated 95% confidence intervals (95% CI) were calculated to estimate VDR genotypes and allele associations with SPTB.

Distribution of numerical variables was tested using the Kolmogorov-Smirnov test. 25(OH)-vitamin D3 serum concentrations were shown as means with standard deviations, whereas differences in concentrations between different groups of participants were analyzed using the *t*-test for independent samples. In addition, differences in 25(OH)-vitamin D3 serum concentrations between different genotypes were tested using one-way ANOVA. In epidemiological characteristics, age, BMI, and birth weight were shown as means and range, and differences between groups were tested using the *t*-test for independent samples. The number of deliveries, gestational age, and birth length were shown as medians and interquartile ranges (IQR), and differences between groups were tested using the Mann–Whitney test.

Multiple regression was used for analyzing the correlations between maternal and newborn 25(OH)-vitamin D3 serum concentrations, as well as the correlation between genotype combinations, 25(OH)-vitamin D3 serum concentrations, and clinical characteristics in different study groups. All tests applied were two-tailed, and a *p* value of 5% or less was regarded as statistically significant.

## 5. Conclusions

The results of our study demonstrate a notable association between the *VDR* Cdx2 gene polymorphism and idiopathic spontaneous preterm birth (ISPTB) in a Caucasian population, adding important insights to the expanding research on genetic factors that affect pregnancy outcomes. These findings are consistent with previous studies that show the role of *VDR* gene variants in 25(OH)-vitamin D3 metabolism and their related health effects, highlighting the complexity of genetic interactions in the context of preterm birth.

Although we have found a link between the Cdx2 polymorphism and ISPTB, the genetic background regulating 25(OH)-vitamin D3 metabolism remains complex and not fully elucidated since it involves different single nucleotide polymorphisms (SNPs) in the *VDR* gene and other related genes. The significant correlation between maternal and preterm infant serum 25(OH)-vitamin D3 levels further shows the importance of maternal 25(OH)-vitamin D3 status in fetal development and pregnancy outcomes. Our study contributes to the available knowledge, which supports the important role of *VDR* gene polymorphisms, especially the Cdx2 variant, in influencing pregnancy outcomes. However, the relatively small sample size analyzed in our study can affect the generalization of our findings. Future research should focus on larger groups of patients and investigate additional *VDR* gene polymorphisms in order to better understand the genetic mechanisms involved in preterm birth. These could lead to targeted interventions and personalized approaches to reducing the risk of neonatal prematurity.

## Figures and Tables

**Table 1:** Description of epidemiological characteristics of patients and controls.

Women	Descriptor	ISPTB Group N = 44	Control Group N = 44	*p*-Value
Years of age	mean (range)	31.5 (17–43)	31.0 (20–42)	0.694 *
Pre-pregnancy BMI (kg/m^2^)		23.9 (18.4–36.4)	22.4 (18.4–31.9)	0.050 *
Number of deliveries	median (IQR)	2 (1–2)	2 (1–2)	0.531 **
Alcohol use	N (%)	0 (0.0)	0 (0.0)	-
Tobacco use		6 (13.6)	11 (25.0)	0.179 ***
Vitamin D supplementation		26 (59.1)	30 (68.2)	-
Previous PTB		4 (9.1)	0 (0.0)	-
PTB in family		10 (22.7)	0 (0.0)	-
**Newborns**				
Gender Male Female	N (%)	27 (61.4) 17 (38.6)	16 (36.4) 28 (63.6)	0.020 ***
Gestational Age (Weeks)	median (IQR)	35 (33–36)	40 (38–40)	*p* < 0.001 **
Birth weight (Grams)	mean (range)	2593.9 (860.0–3400.0)	3565.2 (2750.0–4510.0)	*p* < 0.001 *
Birth length (Centimetres)	median (IQR)	46.5 (33–52)	51.3 (47–57)	*p* < 0.001 **

* *t*-test for independent samples; ** Mann–Whitney test; *** Chi-square test.

**Table 2:** The genotype and allele frequencies of the FokI, ApaI, and Cdx2 variants of the *VDR* gene in preterm and full-term mothers and newborns.

*VDR* Genotype/Allele	Women with ISPTB N (%)	Control Women N (%)	Chi-Square; *p*-Value	ISPTB Newborns	Control Newborns	Chi-Square; *p*-Value
FokI						
CC	17 (38.6%)	14 (31.8%)	2.71; 0.258	18 (40.9%)	13 (29.5%)	2.28; 0.319
CT	19 (43.2%)	26 (59.1%)		16 (36.4%)	23 (52.3%)	
TT	8 (18.2%)	4 (9.1%)		10 (22.7%)	8 (18.2%)	
C	53 (60.2%)	54 (61.4%)	0.02; 0.877	52 (59.1%)	49 (55.7%)	0.21; 0.647
T	35 (39.8%)	34 (38.6%)		36 (40.9%)	39 (44.3%)	
ApaI						
GG	9 (20.5%)	2 (4.5%)	5.09; 0.784	9 (20.5%)	11 (25.0%)	1.17; 0.558
TG	26 (59.0%)	31 (70.5%)		21 (47.7%)	16 (36.4%)	
TT	9 (20.5%)	11 (25.0%)		14 (31.8%)	17 (38.6%)	
G	44 (50.0%)	35 (39.8%)	1.86; 0.173	39 (44.3%)	38 (43.2%)	0.02; 0.879
T	44 (50.0%)	53 (60.2%)		49 (55.7%)	50 (56.8%)	
Cdx2						
AA	5 (11.3%)	8 (18.2%)	1.36; 0.507	0 (0.0%)	10 (22.7%)	17.01; <0.001
AG	12 (27.3%)	14 (31.8%)		19 (43.2%)	24 (54.6%)	
GG	27 (61.4%)	22 (50.0%)		25 (56.8%)	10 (22.7%)	
A	22 (25.0%)	30 (34.1%)	1.75; 0.186	19 (21.6%)	44 (50.0%)	15.45; <0.001
G	66 (75.0%)	58 (65.9%)		69 (78.4%)	44 (50.0%)	

**Table 3:** The 25(OH)-vitamin D3 serum concentrations in patients and control group.

	ISPTB Group (Mean ± SD)	Control Group (Mean ± SD)	*p*-Value *
Women	90.38 ± 41.75	82.36 ± 43.92	0.383
Newborns	48.31 ± 24.82	55.59 ± 30.32	0.221

* *t*-test for independent samples.

## Data Availability

The data presented in this study are available on request from the corresponding author. The data are not publicly available due to GDPR regulations. Data is unavailable due to privacy or ethical restrictions.
